# Deep Learning-Guided Toughening of Irregular Growth-Inspired Metamaterials

**DOI:** 10.3390/ma19214466

**Published:** 2026-10-23

**Authors:** Chuang Liu, Liye Jin, Qing Zhou, Meng Zhao

**Affiliations:** College of Civil Engineering, Nanjing Tech University, Nanjing 211816, China; m19556881180@163.com (L.J.); 19975153291@163.com (Q.Z.); 15552056562@163.com (M.Z.)

**Keywords:** growth-inspired metamaterials, toughness, composite materials, deep learning

## Abstract

Achieving simultaneous enhancement of strength and toughness in composite design remains a long-standing challenge due to their intrinsic trade-off. This study presents a bioinspired, growth-like structural strategy to overcome this limitation in biphasic composite systems. By integrating phase-field fracture modeling with deep-learning-assisted candidate screening, we systematically investigate the influence of growth morphologies on crack propagation, stress distribution, and energy dissipation. Results demonstrate that growth-inspired metamaterials mimicking biological branching and adaptive evolution promote crack deflection and load redistribution without compromising stiffness or strength. A multimodal CNN surrogate, using a structural image and a seven-dimensional vector of building-block proportions as inputs, predicts the complete nominal stress–strain response, from which the elastic modulus, ultimate strength, and volumetric energy absorption are subsequently derived. Compared with the architecture exhibiting the highest volumetric energy absorption in the prescribed 40% soft-phase training subset, the selected architecture exhibits increases of 22.0% in volumetric energy absorption, 35.4% in Young’s modulus, and 39.2% in ultimate strength. These numerical results demonstrate the potential of the proposed framework for efficiently screening biphasic architected composites within the investigated design and loading conditions.

## 1. Introduction

Enhancing structural toughness is a primary topic in materials science, driven by the growing demand for high-performance materials in advanced fields such as aerospace, biomedical devices, and flexible electronics [1,2,3]. In addition, for conditions involving high loading, impact, and cyclic fatigue, materials must simultaneously exhibit high strength and toughness—a combination that remains a fundamental challenge for conventional material systems [4,5,6,7,8,9,10].

Traditional toughening strategies primarily rely on compositional modifications, such as introducing secondary phases or reinforcements to improve fracture resistance. While effective, these approaches are generally constrained by the intrinsic strength–toughness trade-off, where improvements in toughness often come at the expense of strength or stiffness [11,12,13,14,15,16]. Recent efforts have shifted from composition-dominated design toward architecture-dominated paradigms, where mechanical performance is tailored through microstructural architectures that regulate stress distribution and crack propagation [17,18]. In parallel, interface-mediated design strategies have been increasingly explored, where engineered heterointerfaces and phase boundaries are utilized to regulate dislocation activities and suppress crack initiation, thereby enhancing damage tolerance and strength–toughness synergy across multiple length scales [19,20,21]. Furthermore, gradient and hierarchical microstructural designs have been developed to introduce spatial heterogeneity in mechanical properties, enabling progressive deformation and crack-arrest mechanisms that improve fracture resistance under complex loading conditions [22,23,24]. In these approaches, structural geometry plays a central role in enhancing fracture resistance.

Bioinspired structural design offers valuable strategies for overcoming the limitations of conventional materials. Natural biological materials, such as nacre and bone, exhibit exceptional fracture resistance through complex hierarchical architectures that deflect cracks and dissipate energy [25,26,27]. Representative examples include nacre-inspired brick-and-mortar architectures that promote interfacial sliding [28,29,30,31] and Bouligand-type helicoidal architectures, which induce tortuous crack paths through layer misalignment [32,33,34,35,36,37,38]. Despite their effectiveness, most bioinspired designs are based on predefined, static configurations, limiting their adaptability to varying loading conditions [39,40,41]. Recently, growth-inspired metamaterials have emerged as a promising paradigm, mimicking stress-driven branching and proliferation observed in nature [42]. Their irregular, heterogeneous topologies introduce additional degrees of freedom, enabling more effective regulation of crack behavior through deflection, branching, and stress redistribution [43,44,45]. To unlock the potential of such structural irregularity, pioneering efforts have established foundational generative frameworks. For instance, Liu et al. proposed a virtual growth program governed by local probabilistic rules to assemble irregular architected materials from a limited set of building blocks, demonstrating highly programmable macro-mechanical properties and imperfection insensitivity [46]. Expanding on this growth strategy, Jia et al. developed a computational framework to precisely modulate spatial stress distribution toward target values in biological tissue supports using disordered microstructures [47]. More recently, Yang et al. utilized stochastic growth rules to engineer static mechanical cloaking and camouflage within narrow error tolerances, a feat highly challenging for conventional periodic designs [48]. However, while these breakthroughs successfully map the foundational structure–property relationships of irregular metamaterials, their design workflows primarily focus on elastic stress modulation, cloaking, or programmatic morphology generation. The inherently stochastic nature of growth-inspired architectures limits precise control over structural features under extreme loading conditions, hindering reproducibility and quantitative design. As a result, establishing clear structure–property relationships governing progressive damage mechanisms and efficiently exploring the vast design space for dynamic fracture propagation remain significant challenges.

To address these challenges, this study proposes an integrated framework for the design and optimization of growth-inspired biphasic metamaterials. While advanced experimental characterizations, such as full-field Digital Image Correlation (DIC), provide valuable insights into strain localization and non-ideal crack paths in additively manufactured polymers [49], the inherent manufacturing anisotropy and printing-direction dependencies often introduce significant experimental scatter [50], making large-scale physical screening prohibitively expensive. To overcome these empirical limitations, our framework combines growth-inspired structural generation with phase-field fracture modeling—a method capable of representing complex crack evolution without explicit crack tracking [51]—and CNN-based forward prediction for rapid candidate screening. Specifically, a virtual growth program is utilized to generate irregular structural topologies, which are subsequently evaluated within a biphasic phase-field fracture framework to systematically investigate the influence of growth morphologies on crack propagation, stress distribution, and energy dissipation. Under fixed volume fraction constraints, the optimization objective is formulated to maximize volumetric energy absorption, thereby identifying high-performance topologies. Furthermore, a convolutional neural network–based deep learning model is developed as a predictive regression workflow to map structural images directly to their stress–strain responses. The CNN is used as a forward surrogate to rank stochastically generated candidates rather than as a direct geometry-generation or inverse-design model. This methodology provides new insights into architecture-driven toughening and offers a potential computational pathway for screening architected composites.

## 2. Structural Design and Phase-Field Modeling

### Growth-Inspired Metamaterial and Phase-Field Setup

**Figure 1:**
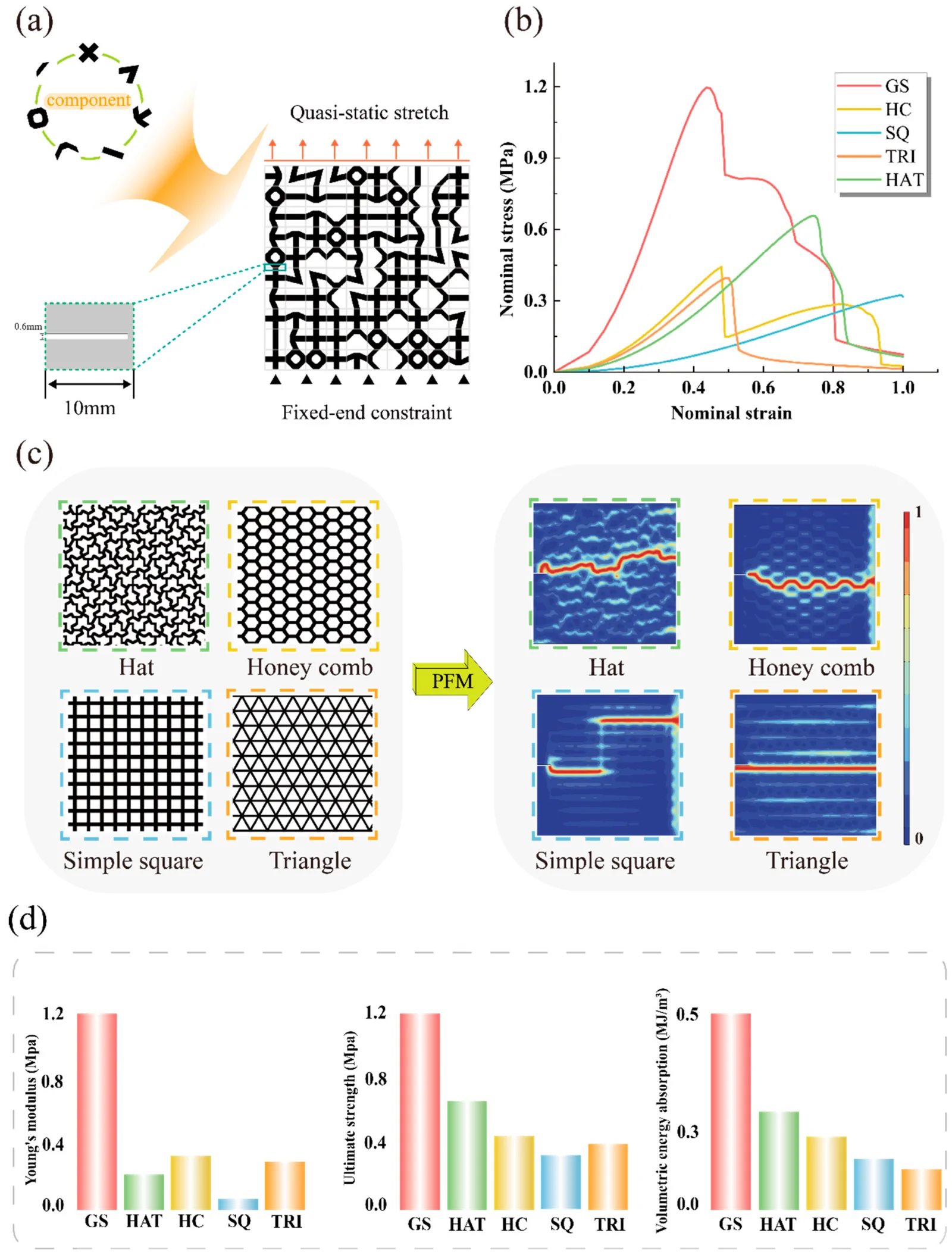
Geometric construction and numerical characterization of the growth-inspired metamaterial. (**a**) Seven predefined building blocks, their assembly within a 10 × 10 discrete design domain, and the applied boundary conditions. Different building-block types are spatially distributed according to prescribed proportion vectors and stochastic growth rules, rather than through periodic repetition of a single unit cell. (**b**) Stress–strain curves obtained after simulating five configurations. (**c**) Simulation results for Hat, HC, SQ, and TRI. (**d**) Comparison of Young’s modulus, ultimate strength and volumetric energy absorption obtained from simulations of all configurations. Note that the growth-inspired (GS) architecture selected here represents a high-performing representative sample used for initial morphological comparison, rather than the absolute optimal configuration of the entire training dataset.

In this study, a growth-inspired metamaterial is defined as an architecture sequentially assembled from a prescribed set of building blocks according to local growth and connectivity rules. Seven predefined building blocks were arranged within a 10 × 10 discrete design domain. At each position, the building-block type was selected according to a prescribed seven-component proportion vector, producing spatially heterogeneous configurations. Here, “irregular” indicates that different building-block types are distributed non-uniformly throughout the domain and that the resulting architecture is not generated by translational repetition of a single unit cell, as occurs in conventional periodic metamaterials. This approach emphasizes structural adaptability, enabling a more favorable balance among toughness, energy dissipation, and overall mechanical properties—without requiring intrinsic enhancements in the constituent material properties. Our study demonstrates that incorporating growth-inspired metamaterials offers distinct advantages for fracture toughening in biphasic composites. In this work, two commercially available photopolymer phases—VeroClear (E = 1000 MPa, v = 0.38, Gc = 3000 J/m^2^) and TangoBlackPlus (E = 0.6 MPa, v = 0.49, Gc = 300 J/m^2^)—were employed as the biphasic material system. All architectures were evaluated using the same 100 mm × 100 mm × 3 mm finite-specimen configuration and identical boundary conditions, ensuring consistent relative comparisons. However, an array-size convergence study was not performed; therefore, the reported responses should be interpreted as specimen-level properties under the present boundary conditions rather than boundary-independent bulk properties. Seven distinct elementary structural motifs were designed and arranged into a 10 × 10 growth-inspired pattern, embedded within a 100 mm × 100 mm × 3 mm square domain, as illustrated in Figure 1a. The geometric generation process combines predefined growth rules with stochastic building-block selection. The design domain and the seven admissible building blocks are specified in advance, whereas the block assigned to each position is randomly sampled according to a prescribed seven-dimensional proportion vector under the implemented growth and connectivity rules. Consequently, different architectures can be obtained from the same proportion vector. No gradient-based optimization or neural network is involved in this geometry-generation stage. The growth-inspired region was composed of the soft phase (TangoBlackPlus), while the surrounding matrix was filled with the stiff phase (VeroClear), thereby establishing a well-defined phase interface.

Previous work on multi-material architected composites using the same VeroClear/TangoBlackPlus photopolymer system has shown that phase-field simulations can reproduce qualitative mechanical trends and complex crack trajectories. Together with the mesh-sensitivity analysis presented in Supplementary Information S1, this evidence supports the use of the present model for controlled relative comparisons among the investigated topologies. However, no direct mechanical validation was performed for the specific architectures studied here. Therefore, the absolute stress and volumetric-energy-absorption values should be interpreted as numerical predictions pending experimental verification.

The initial crack length was 10% of the specimen width (10 mm) with a height of 0.6 mm. Fixed constraints were applied at the bottom while quasi-static tensile loading was imposed at the top; displacement in the x-direction was constrained to zero to eliminate shear components and prevent non-tensile bending moments. For tensile cracking analysis, a linear elastic damage model was adopted. After generating sharp crack geometries, phase-field approximation was applied to embed cracks within the material domain. In the standard energy-driven phase-field formulation employed here, damage evolution is driven by the local elastic strain energy history. Although a strict damage initiation threshold is absent mathematically, stiffness degradation remains negligible during the initial low-strain regime, thereby rigorously preserving the macroscopic linear-elastic response. Damage strictly localizes into distinct cracks only around regions of high stress concentration, rather than uniformly degrading all tensionally loaded areas. The sole material parameter is the critical energy release rate Gc, while tensile strength depends on the damage evolution function and phase-field progression—latter modulated via the internal length scale $l_{int}$, which controls local phase-field width. In this study, $l_{int}$ was set to 0.0015 m, and a staggered solver sequence was employed to enhance computational convergence. In the present model, corresponding to a/W = 0.10 and l_int_/a = 0.15, the conclusions are therefore restricted to this fixed geometric and phase-field scale.

Figure 1b presents the phase-field-simulated nominal stress–strain responses of five representative architectures. All architectures were evaluated using identical material parameters, boundary conditions, and an approximately 40% TangoBlackPlus volume fraction. Comprehensive analysis of the stress–strain curves and corresponding bar charts reveals that the growth-inspired metamaterial (GS) exhibits superior overall mechanical performance. Among the five benchmark architectures compared in Figure 1, the representative GS architecture exhibits the highest Young’s modulus, approximately 1.20 MPa. This indicates a substantially enhanced resistance to deformation during the initial loading stage. As strain increases, GS sustains higher loads and, after reaching peak stress, exhibits a gradual stress decay characterized by multiple plateau-like stages. This behavior effectively mitigates the catastrophic, abrupt failure typical of conventional brittle architectures, highlighting its pronounced ductility and energy absorption capacity. Figure 1c displays the simulation results for the four different architectures.

As illustrated in Figure 1d, the maximum strength of GS approaches 1.2 MPa, comparable to that of the HAT architecture; however, GS demonstrates markedly superior ductility. In contrast, although HAT achieves a high stress peak, it fails at a low fracture strain, exhibiting pronounced brittleness. Regarding volumetric energy absorption, the GS architecture demonstrates a clear advantage, reaching nearly 0.5 MJ/m^3^—significantly outperforming HAT (0.2 MJ/m^3^), HC (0.15 MJ/m^3^), SQ (0.12 MJ/m^3^), and TRI (0.1 MJ/m^3^). Under the prescribed quasi-static loading condition, the higher volumetric energy absorption indicates a greater simulated work-to-failure and improved resistance to progressive damage. While HC exhibits moderate initial stiffness, it suffers from an abrupt post-peak load drop. Similarly, the high-strength HAT architecture fails in a brittle manner. In contrast, SQ and TRI architectures exhibit weak overall performance, particularly suffering from markedly insufficient strength and toughness. The GS architecture, however, achieves an optimal balance through its gradient-like transitions, characterized by gradual post-peak stress decay and enhanced energy dissipation. A mesh-sensitivity analysis using element sizes of 1.5, 1.0, 0.8, and 0.6 mm was conducted at the fixed phase-field internal length *l_int_* = 1.5 mm, corresponding to *h/l_int_* = 1.00, 0.67, 0.53, and 0.40, respectively. With progressive mesh refinement, the nominal stress–strain response, principal crack trajectory, and derived global properties approached stable results, and the responses obtained using the 0.8 and 0.6 mm meshes were nearly coincident. The 1.0 mm mesh reproduced the principal response characteristics and global crack-propagation mode of the finer meshes while reducing the computational cost of generating the 200-sample dataset. It was therefore adopted for the controlled comparisons in this study. Further details are provided in Supplementary Information S1.

## 3. Statistical Analysis and Fracture Mechanisms

### 3.1. Statistical Distribution of Volumetric Energy Absorption

**Figure 2:**
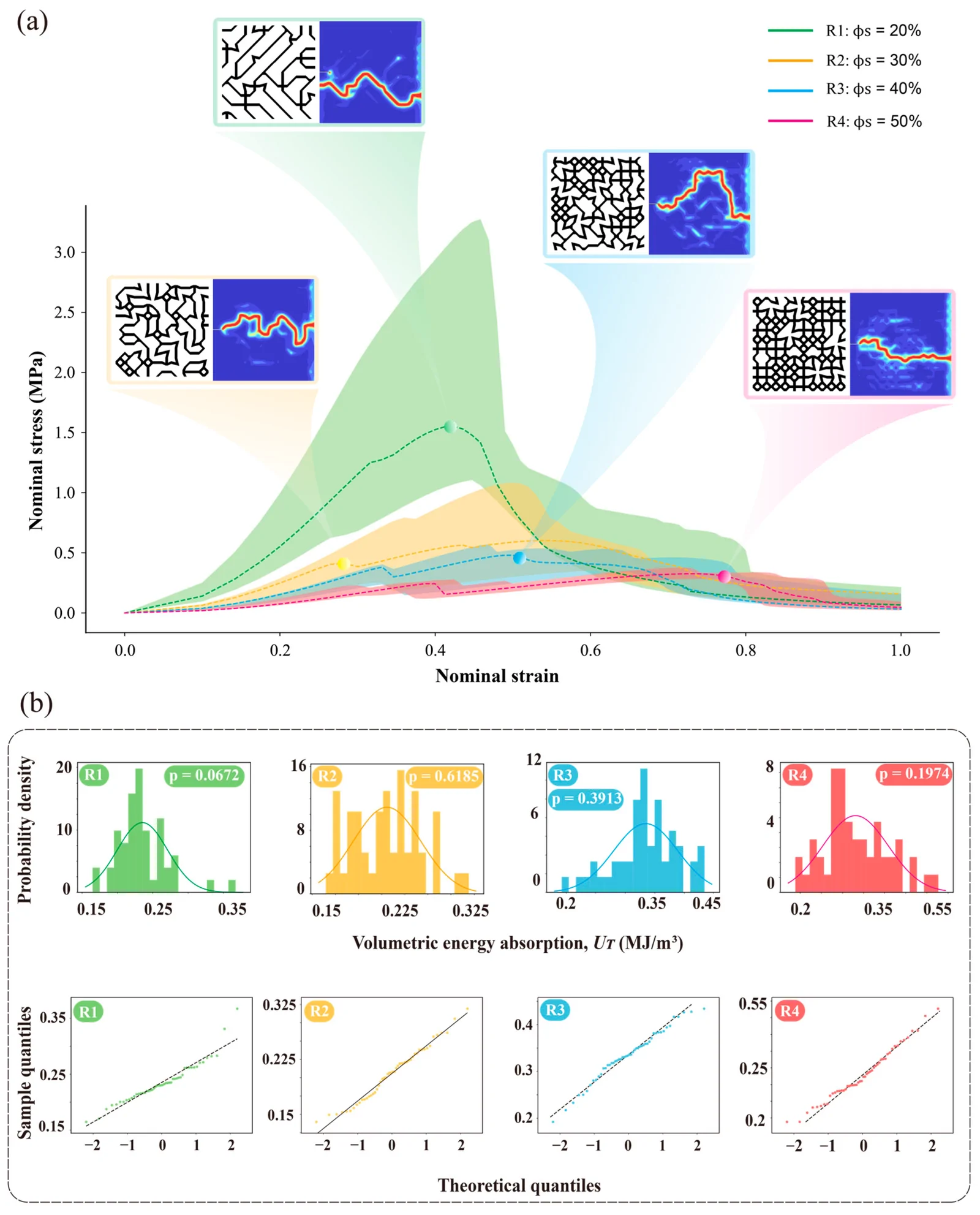
Statistical distributions of the phase-field-simulated mechanical responses. (**a**) Nominal stress–strain responses of growth-inspired architectures with soft-phase volume fractions of 20%, 30%, 40%, and 50%, corresponding to R1, R2, R3, and R4, respectively. Nominal stress is reported in MPa, and nominal strain is dimensionless. (**b**) Probability distributions of volumetric energy absorption *U_T_* in MJ/m^3^ and the corresponding Q–Q plots.

Since the phase ratio in biphasic metamaterials significantly influences their overall mechanical response, four groups of growth-inspired architectures were generated with soft-phase volume fractions of 20%, 30%, 40%, and 50%. These four groups are denoted as R1, R2, R3, and R4, respectively, and are consistently distinguished using the different color coding throughout Figure 2. The dataset used for CNN training and evaluation contained 200 independently generated architectures, including 50 samples for each nominal soft-phase fraction of 20%, 30%, 40%, and 50%. All samples were simulated using the same constituent properties, specimen dimensions, initial crack, boundary conditions, and uniaxial tensile loading. Each data sample consisted of a structural image, a seven-dimensional building-block proportion vector, and the corresponding nominal stress–strain response. Comprehensive stress–strain simulations were conducted for each group, as illustrated in Figure 2a. The results clearly reveal that the stress–strain curves corresponding to different phase ratios not only differ markedly in peak strength but also exhibit systematic shifts in deformation behavior throughout the entire strain history. This shift manifests in two primary aspects: First, as the soft-phase fraction increases, the overall strength of the architecture decreases significantly. For instance, at a soft-phase content of 20%, the architecture exhibits a notably higher stress peak than the other three groups, along with a steeper stress rise during deformation and greater post-peak deformation capacity. In contrast, when the soft-phase fraction reaches 50%, the peak stress drops substantially, and the architecture reaches its peak response, after which damage begins to evolve at a low strain level, demonstrating reduced load-bearing capacity and a markedly shortened elastic regime. Second, with increasing soft-phase content, the post-peak load-retention tendency progressively diminishes, and the stress–strain curves become increasingly flattened. This indicates that the soft phase exerts an increasingly dominant role in the structural response, rendering the composite more prone to post-peak deformation, damage evolution, and premature fracture, thereby making it difficult to sustain high load-carrying capacity and reducing the total energy required for fracture.

The systematic shift among the stress–strain curves indicates a continuous variation in mechanical properties across different biphasic compositions. Although each group contains multiple structural configurations generated with a degree of randomness, their overall responses remain concentrated around a group-specific trend. We therefore test the hypothesis that volumetric energy absorption is statistically consistent with a normal distribution at a fixed biphasic composition. The core of this hypothesis is that while random perturbations in microstructural configuration do influence mechanical performance, they do not alter the macroscopic performance trend governed by the phase ratio; thus, the mechanical responses of different configurations are expected to fluctuate around a common mean. The envelope regions surrounding each main curve in Figure 2a further support the validity of this assumption: although some scatter exists among individual structural samples, the overall trend remains stable, and the variance increases with higher soft-phase content, indicating greater instability in the structural response under soft-phase dominance. It is precisely this regularity in performance dispersion that provides the theoretical foundation for subsequent statistical distribution analysis and normality testing. In summary, variations in the biphasic material ratio significantly influence the overall mechanical performance and deformation mechanisms, leading to a systematic shift in the stress–strain response across different ratio groups. Based on these observations, it is hypothesized that the structural toughness within the same ratio condition follows a normal distribution.

To rigorously evaluate this normality hypothesis, the Lilliefors test was employed. The Lilliefors test is an adaptation of the Kolmogorov–Smirnov (K–S) test, primarily used to assess whether a dataset follows a normal distribution, especially when the population mean and variance are unknown. Its core principle involves comparing the empirical distribution of the sample with the theoretical normal distribution, computing the maximum absolute deviation between them, and using the resulting *p*-value to determine whether the null hypothesis of normality can be rejected. A relatively large *p*-value (typically greater than 0.05) indicates that the deviation from normality is not statistically significant. The key advantage of the Lilliefors test is that it does not require prior knowledge of the mean and standard deviation, making it particularly suitable for the sample sizes used in this study. Four sets of unit proportions were randomly selected (R1, R2, R3, R4), and for each set, 50 structural realizations were generated to calculate volumetric energy absorption as the area under the nominal stress–strain curve. The volumetric energy absorption is defined as:

(1) $$U_{T}=\int_{0}^{\varepsilon_{f}}\sigma_{nom}\left(\varepsilon \right)\;d\varepsilon$$

Here, $U_{T}$ represents the work-to-failure per unit initial volume, or volumetric energy absorption, rather than fracture toughness in the linear-elastic-fracture-mechanics sense. It is distinct from the constituent critical energy-release rate $G_{c}$ used in the phase-field formulation. The unit proportions for these groups are defined by the distribution of the seven building blocks: R1 [0.22, 0.04, 0.05, 0.08, 0.16, 0.20, 0.24], R2 [0.14, 0.16, 0.06, 0.17, 0.11, 0.19, 0.16], R3 [0.06, 0.09, 0.04, 0.32, 0.00, 0.37, 0.12], and R4 [0.26, 0.15, 0.13, 0.25, 0.01, 0.19, 0.01]. The listed proportions were rounded to two decimal places for presentation. Full-precision normalized probabilities satisfying $\sum_{i=1}^{7}p_{i}=1$ were used during structure generation. The upper panel of Figure 2b shows the toughness distributions for each material composition, overlaid with fitted normal distribution curves. The results indicate that most datasets yield relatively high *p*-values—for example, *p* = 0.6185 and *p* = 0.3913—supporting the normality assumption. Even in cases with lower *p*-values (e.g., *p* = 0.0672), they remain greater than 0.05, so the null hypothesis cannot be rejected.

Correspondingly, the lower panel of Figure 2b presents the Q–Q (quantile–quantile) plots. The majority of data points closely follow the 45° reference line, indicating that the data generally satisfy the normality assumption, with only minor deviations in the tails. Such minor discrepancies may arise from localized structural variations or stochastic factors; however, they do not compromise the integrity of the overall statistical patterns. For a detailed explanation, please refer to Supplementary Information S2. Therefore, under a fixed biphasic material ratio, the toughness of different structural realizations is statistically consistent with a normal distribution.

### 3.2. Microstructural Arrangement and Crack Propagation Mechanisms

The statistical results indicate that, for architectures with the same biphasic composition, the distribution of volumetric energy absorption is consistent with normality at the selected significance level. However, empirical data reveal that certain samples exhibit toughness values significantly deviating from the mean. These variations underscore the profound influence of local microstructural arrangement on mechanical performance. For instance, in representative groups such as R1 and R4, although *p*-values (0.0672 and 0.1974, respectively) remain above the conventional significance threshold, the fluctuations highlight how local morphology governs performance. Even with identical phase fractions, differences in spatial distribution lead to pronounced changes in crack propagation paths and global toughness.

**Figure 3:**
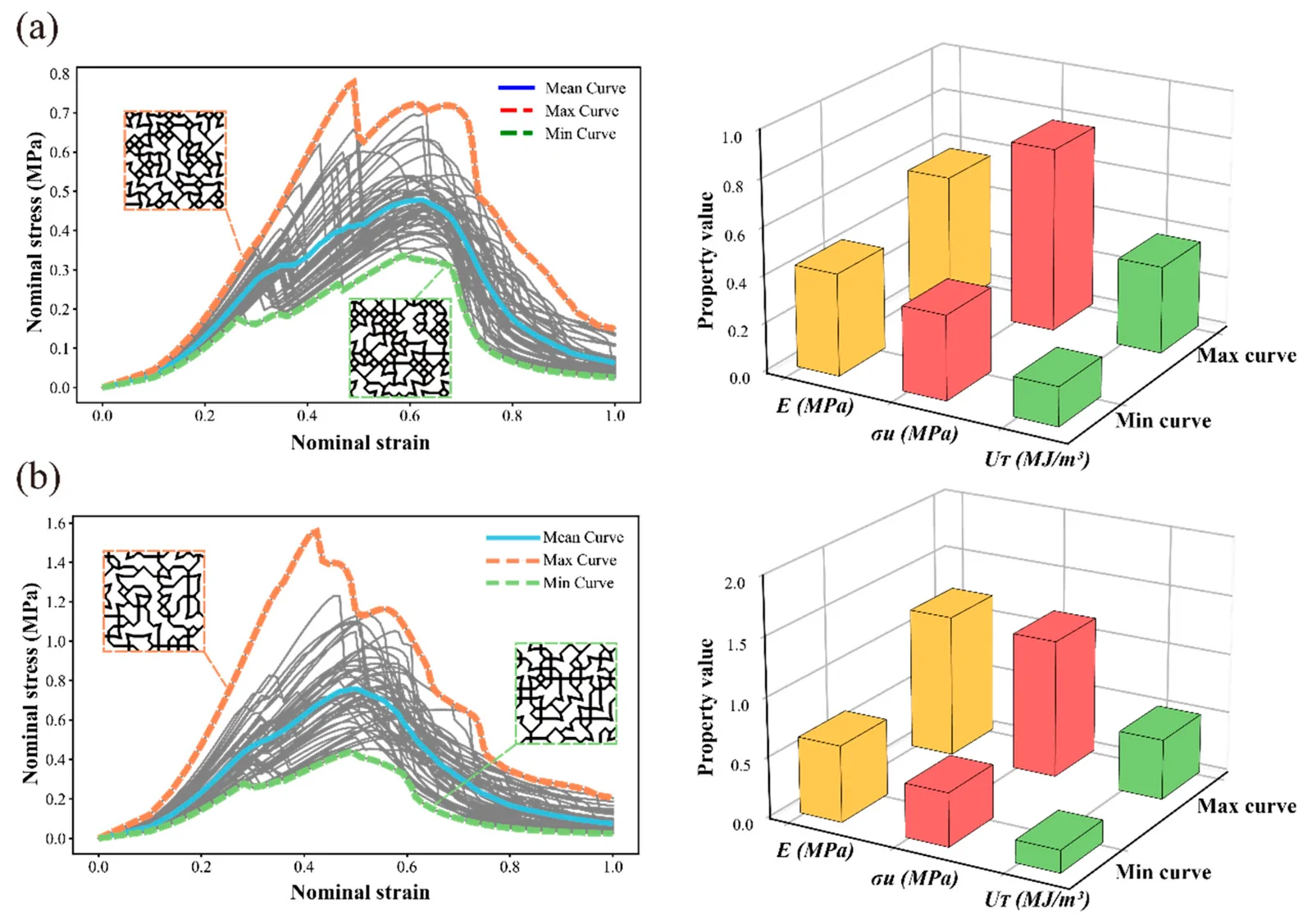
Influence of microstructural arrangement based entirely on phase-field simulations. (**a**) Nominal stress–strain responses and derived properties for representative R1 architectures. (**b**) Corresponding results for representative R4 architectures. Gray curves denote the responses of the individual architectures, whereas the blue, orange, and green curves denote the mean, maximum, and minimum responses, respectively. In the property charts, the yellow, red, and green bars represent *E*, *σ_u_*, and *U_T_*, respectively. Nominal stress, Young’s modulus *E*, and ultimate strength *σ_u_* are reported in MPa; nominal strain is dimensionless; and volumetric energy absorption *U_T_* is reported in MJ/m^3^.

As evident from the stress–strain curves in Figure 3 and the quantitative analysis of crack propagation lengths, subtle differences in the morphology of the growth-inspired metamaterial profoundly influence fracture behavior. In high-performance architectures, cracks repeatedly traverse stiff-phase regions, undergoing multiple deflections and detours. In group R1, the high-performance architecture reaches a crack path of 115.62 mm, compared to 102.50 mm for the low-performance counterpart, which quantitatively demonstrates the increased crack-path tortuosity. An even more stark discrepancy is observed in group R4, where the high-toughness realization reaches 117.50 mm, while the shortest path is only 86.25 mm. This redirection substantially increases the energy required for crack advancement. Cracks are forced to deflect at stiff-phase boundaries, thereby elongating the propagation path and enhancing total energy dissipation.

Enhancement of mechanical response is governed by two complementary mechanisms. Before crack initiation, the connected stiff-phase regions form effective load-transfer paths and redistribute stress away from highly localized regions, thereby improving the initial stiffness and load-bearing capacity. After crack initiation, the heterogeneous soft–stiff interfaces interrupt direct crack propagation and promote crack deflection and branching. The resulting tortuous propagation path delays localized damage coalescence and sustains the post-peak response over a larger strain range, thereby increasing the area under the stress–strain curve. These observations suggest that stress redistribution dominates the pre-peak response, whereas topology-induced crack-path modification provides the primary contribution to post-peak energy dissipation. Further details regarding the material-specific toughening mechanisms and the energetic analysis of these crack-tip interactions are provided in Supplementary Information S3.

The foregoing analysis indicates that while the phase fraction governs the overall trend, the local arrangement is an important factor controlling the upper and lower ranges of mechanical performance. Crack path evolution is an energy-minimization process shaped by the spatial configuration of the stiff phase. This suggests that future design should shift from “proportion control” to “morphology regulation,” considering the hard phase skeleton network and path connectivity. These statistically uncommon cases may represent topological extremes within the sampled growth-generated design space.

## 4. Deep Learning Model and Structural Optimization

### 4.1. Deep Learning Model Architecture and Validation

**Figure 4:**
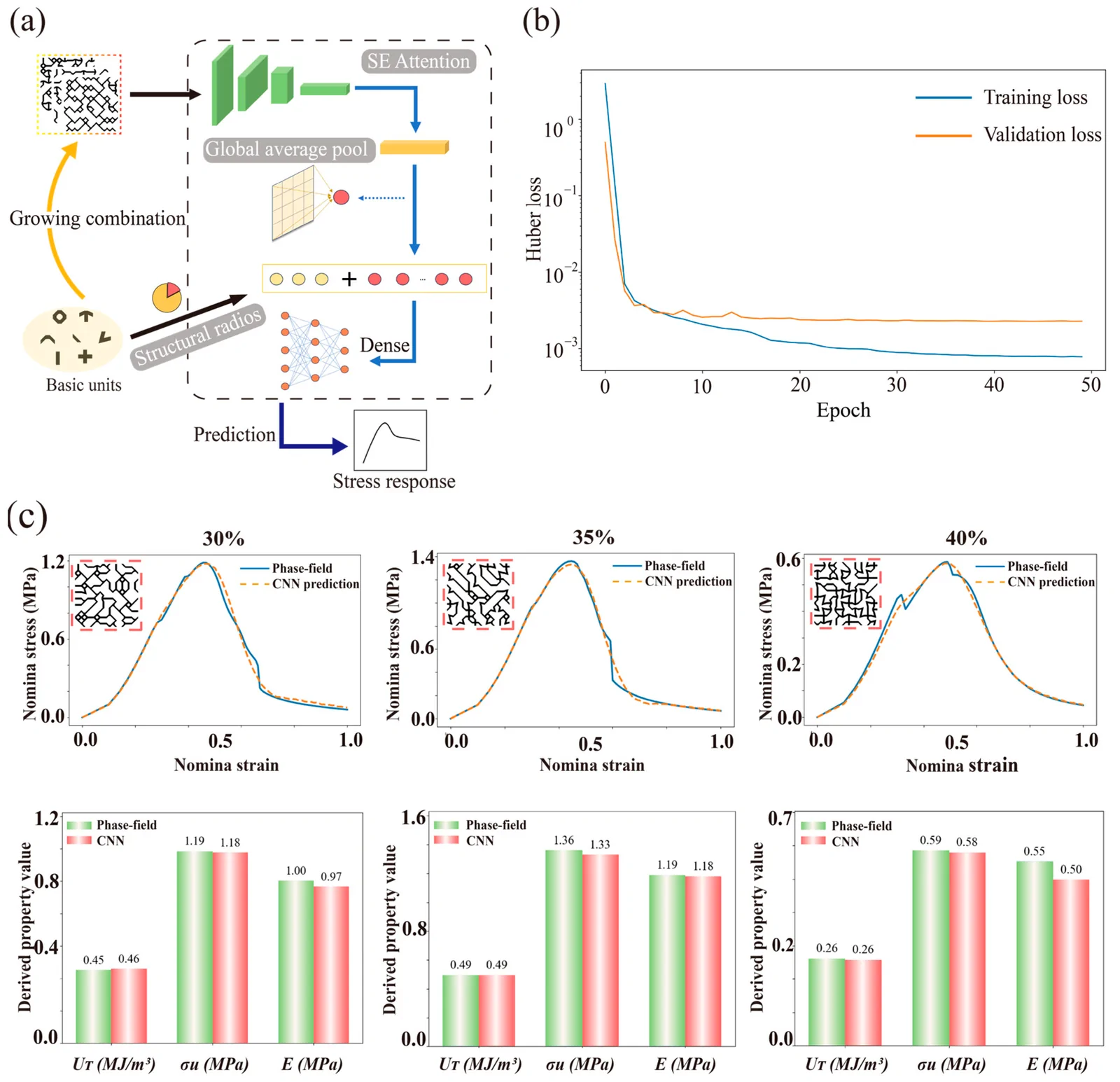
Multimodal network architecture and quantitative validation. (**a**) Architecture of the forward surrogate, which uses a structural image and a seven-dimensional building-block proportion vector to predict the complete nominal stress–strain response. (**b**) Training and validation loss histories. (**c**) Comparison of phase-field references and CNN predictions for representative architectures containing 30%, 35%, and 40% soft phase. Solid curves and green bars denote phase-field results, whereas dashed curves and red bars denote CNN predictions. The three cases in panel (**c**) are presented as representative physical-unit comparisons and do not constitute a statistical assessment of the complete 40-sample validation dataset. The red dashed boxes in panel (**c**) indicate the structural images corresponding to the three representative evaluation architectures.

A multimodal deep regression model was developed to predict the complete nominal stress–strain response of growth-inspired architectures. Using ResNet50 as an automatic feature extractor, the model integrates a custom regression layer to map geometry to mechanical response. The overall architecture is presented in Figure 4a. The input comprises normalized structural images (224 × 224 × 3) and 7-dimensional structural parameters representing unit block composition ratios. These parameters provide explicit global composition information complementary to the spatial features extracted from the structural images. Given the relatively small size of the dataset, training a deep neural network from scratch would be prone to severe overfitting. Therefore, a transfer-learning strategy was explicitly deployed. The image-based representation combined with a CNN leverages robust, generalized feature extractors from pre-trained architectures, reducing the risk of overfitting and facilitating training convergence on the limited dataset. For this data volume, the image-based approach was straightforward to implement and provided usable prediction accuracy within the investigated design domain, so the additional efficiency of a more compact encoding was not a limiting factor. The objective of the present model is to accelerate the prediction and screening of image-based discrete architectures rather than to demonstrate the superiority of the adopted CNN over other machine-learning or topology-optimization methods. Direct phase-field simulations are therefore used as the physics-based reference for evaluating its predictions. The CNN functions strictly as a data-driven forward predictor, while the underlying physical mechanisms are interpreted directly from the independent phase-field simulations. However, if the volume of data becomes extremely large in future studies, selecting more efficient encoding configurations would be necessary to further optimize and improve the computational efficiency. The backbone adopts the ImageNet pre-trained ResNet50 model, retaining the first 49 convolutional layers for multi-scale feature extraction. Following the output of the feature map (7 × 7 × 2048), channel weights are recalibrated via an SE attention module, compressed into a 2048-dimensional vector using global average pooling, and fused with the structural parameters to form a comprehensive representation. The CNN was introduced to reduce the repeated use of phase-field simulations during large-scale candidate screening. Because the building-block identities and their spatial arrangements are discrete variables, the present design space is not naturally represented by the continuous density variables commonly used in gradient-based topology optimization. The trained CNN therefore acts only as a forward surrogate for performance prediction and candidate ranking. The 200 independently generated architectures were divided at the complete-architecture level into 160 training samples and 40 validation samples using an 80:20 stratified split across the four soft-phase groups. Each geometry and its associated structural image, building-block proportion vector, and stress–strain response occurred in only one subset. No rotated, scaled, or otherwise augmented duplicate was distributed across different subsets. All 40 validation architectures were used to calculate the average validation Huber loss at each training epoch, as reported in Figure 4b. The physical-unit comparisons in Figure 4c, however, were conducted only for three representative architectures and should not be interpreted as complete-set error statistics.

The regression head comprises a 512-unit fully connected layer with ReLU activation, a Dropout layer, and a final linear output layer. Unlike conventional scalar-output models, the present architecture directly predicts the complete stress–strain response. Training was performed using the Huber loss and the Adam optimizer, with a batch size of 32 and a maximum of 50 epochs. Transfer learning, L2 regularization, dropout, learning-rate reduction, and early stopping were employed to reduce the risk of overfitting under the limited-data condition. The detailed training settings are provided in Supplementary Information S4. The Huber loss is defined as:

(2) $$L_{\delta }\left(y,\hat{y}\right)=\left\{\begin{array}{ll} \frac{1}{2}{\left(y-\hat{y}\right)}^{2} & if\;\left|y-\hat{y}\right|<\delta \\ \delta \left(\left|y-\hat{y}\right|-\frac{1}{2}\delta \right)\; & otherwise \end{array}\right.$$

where y is the phase-field reference value, $\hat{y}$ is the predicted value, and δ is the turning point. This function ensures robustness by adopting squared loss for small errors and transitioning to linear loss for large errors, mitigating the impact of outliers in physical modeling.

As shown in Figure 4b, both the training and validation losses decrease without a pronounced late-stage divergence. The validation loss in Figure 4b was averaged over all 40 validation architectures at each epoch and therefore characterizes the aggregate model behavior on the complete validation subset in the normalized output space. However, the Huber loss is not directly equivalent to MAE, RMSE, or R^2^ expressed in physical units. Figure 4c provides physical-unit comparisons for three representative architectures containing 30%, 35%, and 40% soft phase. Based on the displayed values, eight of the nine scalar comparisons have relative errors no greater than 3.0%, while the largest relative error is 9.1% for the Young’s modulus of the 40% soft-phase case. These values are provided as illustrative case-level comparisons rather than overall validation-set statistics. Together with the architecture-level data separation, transfer learning, regularization, dropout, and early stopping, this behavior indicates no evident overfitting within the reported training process. Nevertheless, the loss histories alone cannot establish predictive reliability outside the sampled design domain. Figure 4c therefore further compares the CNN predictions with the corresponding phase-field results for three representative evaluation architectures. The 35% soft-phase architecture was generated independently after model training and was used to assess interpolation between the 30% and 40% composition groups.

The corresponding MAE/RMSE values are 0.0033/0.0058 MJ/m^3^, 0.0167/0.0191 MPa, and 0.0300/0.0342 MPa, respectively, as summarized in Table 1. Because these statistics are based on only three representative evaluation architectures, they should be interpreted as descriptive validation within the investigated design domain rather than evidence of broad extrapolative generalization or calibrated probabilistic uncertainty.

### 4.2. Structural Optimization and Performance Enhancement

Enhancing energy dissipation by maximizing volumetric energy absorption is a central objective of the present candidate-screening task. Following the training of the predictive model, the CNN was applied to a large-scale candidate-screening task to identify high-performing micro-topologies under the prescribed biphasic composition. To reduce the influence of composition-driven variations and examine topology-induced toughening, the optimization task was formulated as a conditional optimization problem under a prescribed material budget constraint. Specifically, the soft-phase fraction $\phi_{s}$ was restricted to a narrow target range of $\phi_{s}=0.40\pm 0.02$, which directly matches the baseline architectures evaluated in Growth-Inspired Metamaterial and Phase-Field Setup Section to serve as a representative case study. The optimization objective is formulated as: $\underset{\theta }{\max}U_{T}\left(\theta \right)$ subject to $|\phi_{s}\left(\theta \right)-0.40|\leq 0.02$, where $U_{T}$ represents the volumetric energy absorption calculated from the nominal stress–strain curve and θ denotes the micro-topological configuration vector. This conditional formulation focuses on maximizing mechanical performance within a narrow prescribed composition range rather than searching for a globally optimal phase fraction. We randomly generated 10,000 growth-inspired configurations using the same virtual growth rules and employed the trained CNN only as a forward surrogate for rapid performance prediction and candidate ranking. The CNN did not directly generate or modify the geometries. This surrogate-assisted prediction evaluated 10,000 candidates in merely a few seconds, a task that would be computationally prohibitive if relying on direct phase-field fracture simulations.

**Figure 5:**
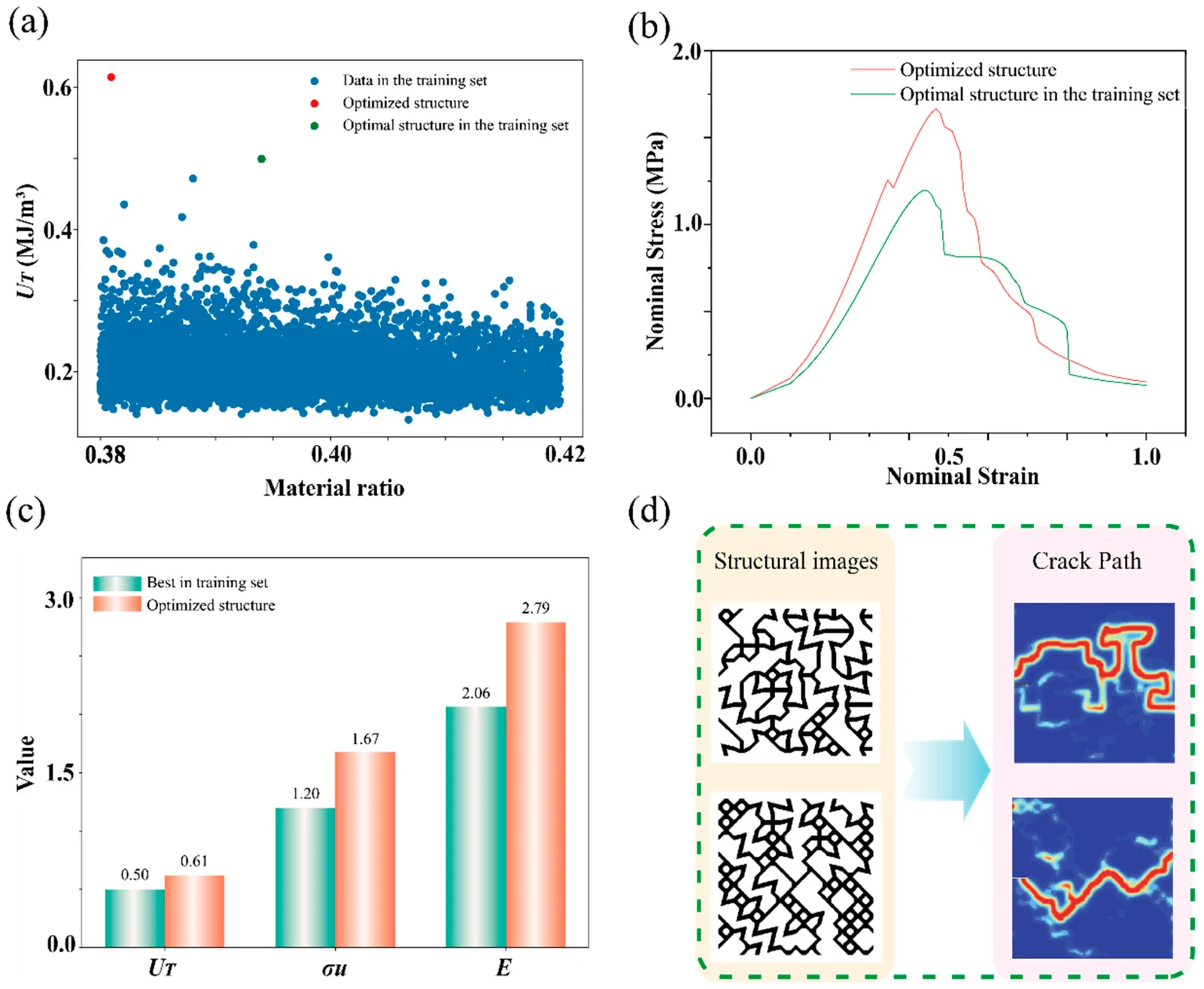
Optimization results. (**a**) Comparison of the toughness between the optimized architecture and the training set. (**b**) Comparison of the stress–strain curves between the optimized architecture and the optimal architecture in the training set. (**c**) Comparison of the Young’s modulus, ultimate strength, and volumetric energy absorption between the optimized architecture and the best in the training set. (**d**) Representative structural images and phase-field-predicted crack paths. The leftmost photograph shows a printed geometry demonstrator only; no mechanical test data are reported. The CNN was used for candidate ranking, whereas all quantitative mechanical responses reported here were obtained from independent phase-field simulations of the selected configurations.

As illustrated in Figure 5a, the selected architecture significantly surpasses all samples in the training set in terms of toughness. Notably, the soft-phase proportion in the optimized individual is 38.09%, while the best-performing architecture in the training set contains 39.40%. As shown in Figure 5c, the optimized candidate contains $\phi_{s}=38.09\%$ soft phase, while the training-set baseline contains $\phi_{s}=39.40\%$. Both configurations satisfy the prescribed material budget constraint of $\phi_{s}=0.40\pm 0.02$. Despite a difference of 1.31 percentage points in soft-phase fraction, the selected architecture achieves a volumetric energy absorption of 0.61 MJ/m^3^, compared with 0.50 MJ/m^3^ for the training-set reference, corresponding to an improvement of 22%. Although the effects of composition and topology cannot be strictly separated using these two architectures, their comparable soft-phase fractions suggest that micro-topological characteristics, including stiff-phase network connectivity and crack-path tortuosity, contribute substantially to the improved mechanical response.

The combined nominal stress–strain response in Figure 5b and the representative phase-field damage and stress distributions in Figure 5d provide evidence for the transition from pre-peak load redistribution to post-peak crack-path modification. Compared with the training reference, the selected architecture contains a more continuous stiff-phase load-transfer network, which alleviates severe stress localization and promotes stress redistribution during the pre-peak loading stage. After crack initiation, the irregular soft–stiff interfaces interrupt direct crack propagation and promote crack deflection and branching along multiple local paths, thereby extending the effective propagation path and delaying localized damage coalescence. Consistent with these observations, the selected architecture exhibits a Young’s modulus of 2.79 MPa, representing a 35.4% increase relative to the training-reference value of 2.06 MPa, as detailed in Table 2. The nominal stress–strain curves in Figure 5b further show that the selected architecture reaches a peak stress of 1.67 MPa, compared with 1.20 MPa for the training reference, corresponding to an increase of 39.2%. It also maintains a more gradual post-peak response over an extended strain range. Consequently, the volumetric energy absorption, calculated as the area under the nominal stress–strain curve, increases from 0.50 to 0.61 MJ/m^3^, corresponding to an improvement of 22.0%. These results indicate that the improved pre-peak load transfer and topology-induced modification of the crack path jointly contribute to the enhanced mechanical response.

The physics-based origin of the simultaneous improvement in elastic modulus, ultimate strength, and volumetric energy absorption lies in the synergistic effect of load redistribution and topology-induced crack deviation. In conventional periodic architectures, structural symmetry often results in straight crack paths and severe localized stress concentrations under specific loading conditions, leading to early failure. In contrast, the irregular, growth-inspired topologies inherently lack translational symmetry. This spatial non-periodicity improves material utilization by distributing the structural load more uniformly across the interconnected microstructural network, thereby enhancing both the macroscopic stiffness and strength before damage initiation. Concurrently, during the progressive damage process, this growth-inspired topology forces the propagating crack to undergo continuous deflection and branching along the complex soft–hard phase interfaces. This topology-driven crack path tortuosity significantly increases the total fracture surface area and subsequent phase-field energy dissipation. Consequently, the growth strategy improves the balance among stiffness, ultimate strength, and volumetric energy absorption through a combined mechanism of distributed load-bearing in the initial regime and multi-path dissipation during fracture propagation. In summary, these numerical findings indicate that structural reorganization can improve mechanical performance within the investigated design domain even at similar material ratios. However, the performance advantage identified here is based on idealized geometries and has not yet been statistically verified under realistic manufacturing tolerances. While random sampling across 10,000 architectures yielded significant gains, future work could integrate generative adversarial networks (GANs) with the predictive model to enable direct inverse design, efficiently navigating the design space to maximize volumetric energy absorption.

## 5. Conclusions

This study establishes an integrated computational framework combining growth-inspired structural design, phase-field fracture modeling, and deep-learning-assisted candidate screening to investigate the mechanical and fracture responses of biphasic metamaterials. By systematically varying phase ratios and exploring irregular growth morphologies, the relationship between structural configuration and mechanical response was quantitatively analyzed. The findings are restricted to the quasi-static loading regime, and rate-dependent viscoelastic effects of the photopolymers are not considered. The present simulations assume idealized geometries and homogeneous constituent properties; consequently, the effects of dimensional deviations, surface roughness, local voids, material variability, and printing-induced anisotropy are not included and require further investigation. Direct mechanical validation of the specific architectures investigated here was not performed. Consequently, the absolute stress and volumetric-energy-absorption values should be interpreted as numerical predictions, and their practical performance requires future verification under matched specimen dimensions, material calibration, fabrication conditions, fixture constraints, loading rate, and full-field measurement conditions.

The numerical results show that, although phase ratio governs the overall mechanical trend, microstructural arrangement substantially influences the upper range of volumetric energy absorption at a prescribed composition. Under fixed-composition conditions, the distributions of volumetric energy absorption are statistically consistent with normal distributions, while selected high-performing topologies can substantially exceed the corresponding group averages. Architectures with higher volumetric energy absorption exhibit pronounced crack deflection, branching, and delayed damage coalescence associated with interconnected stiff-phase networks, leading to enhanced post-peak energy dissipation. The training and validation loss histories show no pronounced late-stage divergence, and the three representative cases in Figure 4c exhibit satisfactory agreement between the CNN predictions and phase-field references. However, a complete physical-unit error analysis over the entire validation subset was not performed, and the demonstrated use of the model remains limited to candidate screening within the investigated design domain. These results support the use of the model for rapid candidate screening within the investigated design domain. However, because the model was developed using only 200 architectures generated from one building-block set and one family of growth rules, its demonstrated predictive capability is limited to interpolation within the sampled design space and should not be interpreted as broad extrapolative generalization. Within the prescribed 40% ± 2% soft-phase range, phase-field verification indicates that the selected architecture achieves numerical increases of 22.0% in volumetric energy absorption, 39.2% in ultimate strength, and 35.4% in Young’s modulus relative to the explicitly defined training-set reference. These numerical results indicate that growth-inspired metamaterials have substantial potential to improve the balance among stiffness, strength, and volumetric energy absorption under prescribed material-composition constraints. The proposed framework therefore provides a potential computational strategy for screening complex material architectures within the investigated quasi-static loading conditions, rather than establishing experimentally verified manufacturing-scale performance. Furthermore, owing to the non-uniqueness of the geometry-to-property mapping, exploring geometrically diverse but mechanically equivalent architectures and evaluating their robustness under non-uniaxial loading conditions, such as shear and bending, remain important objectives for future work.

## Figures and Tables

**Table 1:** Descriptive error metrics for the three representative architectures shown in Figure 4c.

Mechanical Property	MAE	RMSE	R^2^	Maximum Relative Error
Elastic modulus	0.0300	0.0342	0.9838	9.1%
Ultimate strength	0.0167	0.0191	0.9966	2.2%
Volumetric energy absorption, *U_T_*	0.0033	0.0058	0.9967	2.2%

Note: These metrics were calculated only from the three representative architectures shown in Figure 4c and should not be interpreted as the overall prediction accuracy of the complete 40-sample validation dataset.

**Table 2:** Comparison of mechanical properties between the optimized architecture and the matched-composition training-set baseline.

Mechanical Property	Optimized Architecture	Training-Set Baseline	Enhancement
Elastic modulus	2.79 MPa	2.06 MPa	35.4%
Ultimate strength	1.67 MPa	1.20 MPa	39.2%
Volumetric energy absorption, *U_T_*	0.61 MJ/m^3^	0.50 MJ/m^3^	22.0%

## Data Availability

The original contributions presented in this study are included in the article/Supplementary Material. Further inquiries can be directed to the corresponding author.

## Supplementary Materials

The following supporting information can be downloaded at: https://www.mdpi.com/article/10.3390/ma19214466/s1.
