# Supplementary material for: Deep Learning-Guided Toughening of Irregular Growth-Inspired Metamaterials
Source: Materials (Basel). 2026 Oct 23;19(21):4466. doi: 10.3390/ma19214466 (PMC13763590; doi:10.3390/ma19214466)
Supplement: Supplementary file 1 [file materials-19-04466-s001.zip › materials-4629245-supplementary.pdf]

Supporting information

Deep learning-guided toughening of irregular growth-inspired metamaterials

Chuang Liu[a,*], Liye Jin[a], Qing Zhou[a], Meng Zhao[a]

[a]College of Civil Engineering, Nanjing Tech University, Nanjing, Jiangsu 211816, China

*Corresponding author, Email: liuc@njtech.edu.cn

S1. Mesh sensitivity and accuracy analysis

Accurate phase-field analysis and robust prediction of material response typically require high-resolution mesh refinement in the vicinity of propagating cracks. Such refinement enhances the resolution of the stress field near the crack tip and enables more faithful capture of crack propagation trajectories. However, in this study, the introduction of growth-inspired structures leads to a critical complication: the soft phase (TangoBlackPlus) adjacent to the initial crack may undergo localized fracture either prior to or concurrently with the propagation of the primary crack under tensile loading. This unexpected local cracking behavior implies that mesh refinement confined solely to the initial crack region may fail to adequately resolve the full crack evolution process, thereby compromising the accuracy of the phase-field simulation.

In this study, given the need to generate a large dataset for subsequent neural network training, a uniform mesh discretization was applied across the entire domain rather than employing localized refinement solely around the crack region, while still ensuring adequate accuracy in crack simulation. Specifically, the maximum element size was set equal to l_{int}, a scale considered to represent the upper bound of a reasonable mesh resolution for phase-field modeling. This strategy offers two key advantages: first, it enables robust simulation of concurrent crack initiation and propagation at multiple sites, avoiding numerical artifacts arising from excessively coarse or overly refined local meshes; second, compared to adaptive or hierarchical mesh refinement approaches, it substantially reduces computational cost during dataset generation, thereby enhancing overall computational efficiency. Naturally, if computational resources permit and higher fidelity in crack evolution is required, a finer mesh can be adopted to more accurately resolve the spatial gradients of the phase-field variable, thus improving the stability of predicted material responses. To ensure the validity of the numerical simulations and to strike a rational balance between accuracy and computational expense, a mesh sensitivity study was conducted using four element sizes—1.5 mm, 1.0 mm, 0.8 mm, and 0.6 mm—to evaluate their influence on key mechanical parameters. Comparative analyses of stress–strain curves, crack propagation paths, Young's modulus, ultimate strength, and toughness indicate that, within a certain range, variations in mesh resolution do not significantly alter the global crack propagation pattern, although they may affect local numerical features to

some extent.

As illustrated in Figure S1(a), the comparison of stress - strain curves obtained under four mesh densities reveals similar overall trends across all cases—namely, an initial elastic region, a distinct yield peak, followed by a stress-softening stage. This indicates that variations in mesh density do not substantially alter the fundamental mode of crack propagation. However, the coarser mesh (1.5 mm) exhibits a slightly higher stress peak compared to the finer meshes (0.6 mm and 0.8 mm), suggesting that coarser discretization may moderately overestimate the apparent stiffness of the material. This behavior likely stems from the reduced degrees of freedom in crack path evolution associated with larger elements, which promotes a more uniform stress distribution and consequently delays the onset of localized stress concentration. As mesh refinement increases, the peak stress gradually decreases and approaches convergence; notably, the curves for 0.8 mm and 0.6 mm mesh sizes nearly overlap, indicating that further mesh refinement within this range yields negligible improvements in computational accuracy.

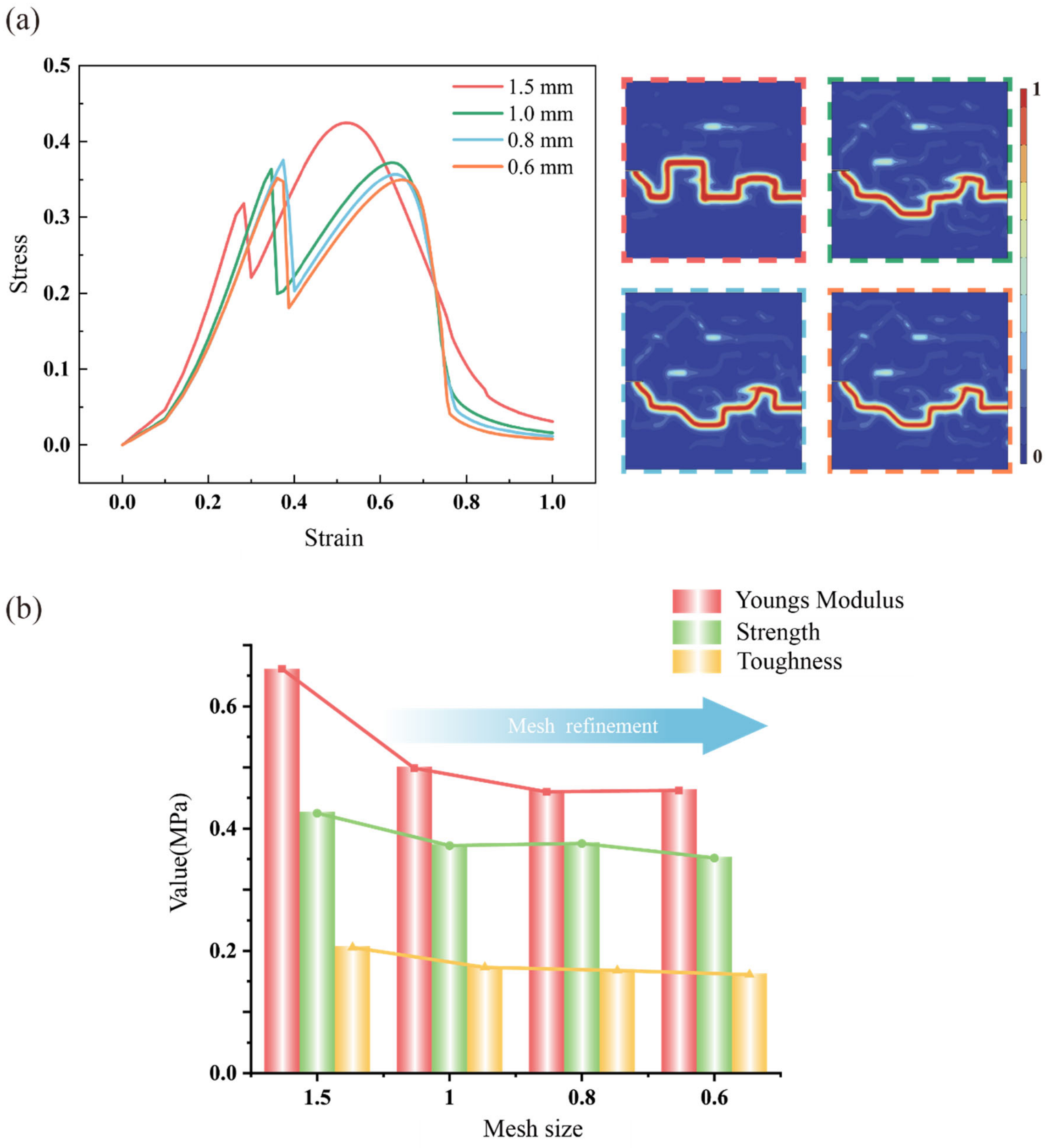

S1. Mesh-sensitivity analysis at the fixed phase-field internal length l_{int}=1.5 mm. The element sizes of 1.5, 1.0, 0.8, and 0.6 mm correspond to h/l_{int}=1.00, 0.67, 0.53, and 0.40, respectively. (a) Stress-strain curves and simulation results for different mesh sizes. (b) Variations in Young's modulus, strength, and toughness with mesh size.

The results of crack propagation paths further reveal that, although the overall trajectories are consistent across different mesh resolutions, the coarser mesh produces slightly smoother crack-tip morphologies, whereas finer meshes capture finer microscale features of crack advancement more accurately. This enhanced resolution likely stems from

the improved ability of high-resolution discretization to resolve subtle fluctuations during crack propagation. Nevertheless, this gain in local detail does not lead to any substantial deviation in the global crack path, indicating that further mesh refinement in the present simulations primarily affects local crack morphology rather than the overall fracture trend. As illustrated in Figure S1(b), the calculated Young's modulus values exhibit minor discrepancies between the coarse and fine meshes; however, these variations remain well within an acceptable error margin. Similarly, the ultimate strength—defined as the material 's maximum load-bearing capacity—shows negligible variation across mesh resolutions, confirming that mesh size has little influence on the predicted load-carrying performance. Furthermore, toughness, quantified as the area under the stress–strain curve, demonstrates consistent integral values across all mesh sizes, as the overall shape of the stress–strain response remains largely unchanged. This indicates that adjustments in mesh resolution have limited impact on the predicted energy absorption capacity of the material.

In phase-field modeling of fracture, the characteristics of crack propagation are primarily governed by the internal length scale l_{int}, a key model parameter that regularizes the crack. Consequently, the mesh size must be sufficiently fine to resolve the diffuse crack zone, yet not excessively refined to avoid prohibitive computational costs. When the element size is on the order of l_{int}, the computed crack path achieves numerical convergence, and further mesh refinement yields negligible improvements in accuracy. In this study, the maximum element size was set equal to l_{int}, ensuring that the mesh resolution is commensurate with the intrinsic crack width. This choice preserves the physical fidelity of crack evolution while maintaining numerical stability. If the mesh is too coarse, the accuracy of the crack trajectory deteriorates, potentially leading to erroneous propagation modes. Conversely, an overly fine mesh can result in excessively steep phase-field gradients, inducing numerical instabilities that compromise solution convergence.

Taking both computational accuracy and efficiency into account, a mesh size of 1.0 mm was ultimately selected for all simulations. Although finer meshes can offer marginally higher resolution, they incur substantially increased computational costs. The 1.0 mm mesh, however, adequately captures the essential features of the global fracture process, with numerical errors remaining within an acceptable range. Therefore, under the premise of

ensuring data validity, this mesh size strikes an optimal balance between accuracy and computational efficiency, making it well-suited for the subsequent generation of large-scale datasets and training of machine learning models.

S2. Analysis of the reasons for minor point deviations in Q-Q plots

This trend can be explained by the microstructural characteristics of the material. In this study, the biphasic composite is composed of seven elementary building blocks—corner, cross, line, skew, T, V, and X—each of identical size. Within each unit, the black regions represent the soft matrix, while the remaining regions correspond to the stiff matrix. These units are randomly arranged under the constraint of maintaining global structural connectivity. Although each realization yields a distinct configuration, the overall phase composition remains fixed when the biphasic ratio is held constant. Since macroscopic properties typically emerge as statistical averages of microstructural features, local morphological differences are smoothed out by the collective effect when a sufficiently large number of randomly assembled units are used, ultimately leading to a stable statistical distribution. This behavior is analogous to the central limit theorem, which states that the distribution of the sum (or average) of a large number of independent random variables tends toward normality. Therefore, under a fixed biphasic material ratio, the toughness of different structural realizations—despite their geometric differences—still follows a normal distribution trend.

S3. Material-specific toughening mechanisms and the energetic analysis

To supplement the numerical and morphological analysis in the main text, the mechanistic roles of the constituent materials are detailed as follows:

Stiff Phase (VeroClear): Functions as a primary “deflector.” Its high fracture energy barrier induces the tortuosity observed in the high-performance R1 and R4 samples.

Soft Phase (TangoBlackPlus): Facilitates global compliance but, when forming continuous channels, leads to straight, low-energy fracture paths (e.g., 86.25 mm in R4).

Energetic Retardation: High-toughness architectures exhibit a higher energy release rate retardation capability. The irregular distribution of stiff phases interferes with the driving force at the crack tip, ensuring that the total work done during fracture (the area

under the stress – strain curve) is maximized through path elongation and interfacial energy dissipation.

S4. Technical training parameters and numerical validation training details

It incorporates L2 regularization ($\lambda = 0.01$) and a 50% Dropout probability. The Adam optimizer has an initial learning rate of 1×10^{-4} and employs the "Reduce LR on Plateau" strategy (if the validation loss does not improve for 5 consecutive epochs, the learning rate is reduced by 50%). Additionally, an early stopping mechanism is implemented (training is terminated if the validation loss does not improve after 10 epochs). The training/validation data is randomly divided in a stratified manner with a ratio of 8:2, and the training batch size is set to 32 to ensure a balance between GPU memory utilization and gradient update efficiency. Validation Data: For the 30% soft substrate sample, the predicted toughness is 0.46 MPa (Actual: 0.45 MPa, ~2% error), ultimate stress is 1.18 MPa (Actual: 1.19 MPa, <1% error), and the elastic modulus is 0.97 MPa (Actual: 1.0 MPa). These narrow error margins across all test groups (30%, 35%, 40%) confirm the model’s high precision.
